# Microbial pigments: learning from the Himalayan perspective to industrial applications

**DOI:** 10.1093/jimb/kuac017

**Published:** 2022-08-06

**Authors:** Subhash Kumar, Vijay Kumar, Ambika, Deepika Nag, Virender Kumar, Sanyukta Darnal, Vikas Thakur, Vijeta Patial, Dharam Singh

**Affiliations:** Biotechnology Division, CSIR-Institute of Himalayan Bioresource Technology, Palampur, Himachal Pradesh 176 061, India; Academy of Scientific and Innovative Research (AcSIR), Ghaziabad-201002, India; Biotechnology Division, CSIR-Institute of Himalayan Bioresource Technology, Palampur, Himachal Pradesh 176 061, India; Biotechnology Division, CSIR-Institute of Himalayan Bioresource Technology, Palampur, Himachal Pradesh 176 061, India; Academy of Scientific and Innovative Research (AcSIR), Ghaziabad-201002, India; Biotechnology Division, CSIR-Institute of Himalayan Bioresource Technology, Palampur, Himachal Pradesh 176 061, India; Biotechnology Division, CSIR-Institute of Himalayan Bioresource Technology, Palampur, Himachal Pradesh 176 061, India; Biotechnology Division, CSIR-Institute of Himalayan Bioresource Technology, Palampur, Himachal Pradesh 176 061, India; Academy of Scientific and Innovative Research (AcSIR), Ghaziabad-201002, India; Biotechnology Division, CSIR-Institute of Himalayan Bioresource Technology, Palampur, Himachal Pradesh 176 061, India; Academy of Scientific and Innovative Research (AcSIR), Ghaziabad-201002, India; Biotechnology Division, CSIR-Institute of Himalayan Bioresource Technology, Palampur, Himachal Pradesh 176 061, India; Academy of Scientific and Innovative Research (AcSIR), Ghaziabad-201002, India; Biotechnology Division, CSIR-Institute of Himalayan Bioresource Technology, Palampur, Himachal Pradesh 176 061, India; Academy of Scientific and Innovative Research (AcSIR), Ghaziabad-201002, India

**Keywords:** Himalaya, Microbial ecology, Microbial pigments, Bioprocess, Industrial biotechnology

## Abstract

Pigments are an essential part of life on earth, ranging from microbes to plants and humans. The physiological and environmental cues induce microbes to produce a broad spectrum of pigments, giving them adaptation and survival advantages. Microbial pigments are of great interest due to their natural origin, diverse biological activities, and wide applications in the foods, Pharmaceuticals, cosmetics, and textile industries. Despite noticeable research on pigment-producing microbes, commercial successes are scarce, primarily from higher, remote, and inaccessible Himalayan niches. Therefore, substantial bioprospection integrated with advanced biotechnological strategies is required to commercialize microbial pigments successfully. The current review elaborates on pigment-producing microbes from a Himalayan perspective, offering tremendous opportunities for industrial applications. Additionally, it illustrates the ecological significance of microbial pigments and emphasizes the current status and prospects of microbial pigment production above the test tube scale.

## Introduction

The solar photon owns the diversity of colors/pigments on earth in the visible spectrum. Pigment production results from a complex interaction of a cell/organism with its environment (Cuthill et al., 2017). Microbes, including bacteria, produce various pigments with diverse physicochemical and ecological functions (Narsing Rao et al., 2017; Chatragadda & Dufossé, 2021). The primary function of pigments in plants is to harvest solar energy. Similarly, microbial pigments help cells in photoprotection, defense, community-level interactions, and competition, with many aspects yet to be discovered (Silva et al., 2021). The diversity in structure and functions of microbial carotenoids (utilizing light energy, neutralizing oxidants, and role as virulence factors) is another prominent example (Supplementary Fig. S1) (Nupur et al., 2016).

**Fig. 1.**
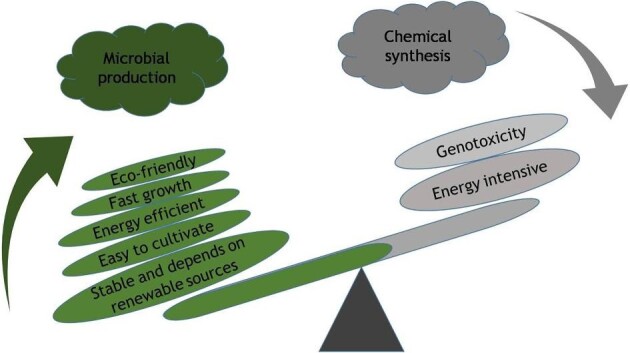
Advantage of microbial production of pigments over chemical synthesis.

Eukaryotes and prokaryotes produce pigments for numerous purposes in different capacities. Plants produce a variety of pigments (Carvalho et al., 2011), but they have several drawbacks, including non-availability, scalability, stability, content, and impurities (Usman et al., 2017). In contrast, microbial pigments are devoid of such limitations and serve as a readily available source of important natural biomolecules (Narsing Rao et al., 2017). Other benefits of microbial production include renewable sources and superior quality product formation compared to chemical synthesis (Thakur et al., 2016). Synthetic dyes and pigments have various health and environmental concerns. Few FDA-approved synthetic dyes used in food, pharmaceuticals, and cosmetic preparation resulted in health-related and environmental issues. For example, sunset yellow and tartrazine result in allergic effects, benzidine dyes result in bowel cancer, and carbon black, widely used as printing ink, is also a potential carcinogen (Narsing Rao et al., 2017). In addition, unethical and untreated discharge of industrial dye effluents produces toxic compounds and persists longer in the environment (Babitha, 2009). Therefore, microbial pigments are preferred over their chemical counterparts. Added advantages are microbial pigment's ease of production and processing supplemented with diversified biological activities, such as antimicrobial, anticancer, antioxidant, and antituberculosis (Chatragadda & Dufossé, 2021; Chen et al., 2021; Silva et al., 2021). Different bacterial pigments with potential bioactivities have been summarized elsewhere (Venil et al., 2020; Celedón and Díaz, 2021). Therefore, it is not discussed in detail in the current review article. However, a brief comparative account of microbial pigment production over chemical synthesis of pigments is illustrated in Fig. 1.

Microbial pigments and colors are important for various applications such as food, clothing, housing, and other commodities (Narsing Rao et al., 2017; Finger et al., 2019; Ramesh et al., 2019; Sen et al., 2019; Chatragadda & Dufossé, 2021). The demand for natural colors is exponentially increasing due to the harmful effects of synthetic dyes. The worldwide pigment market is valued at over USD 32.9 billion in 2020 and is further projected to grow at a CAGR of over 5.1% during the forecast period (2021–2028) (https://www.grandviewresearch.com/industry-analysis/dyes-and-pigments-market). The global pandemic of COVID-19 has significantly affected the dyes and pigments market in the past 2 years. During the period, the prohibition of construction works negatively impacted the global paint industry. Nevertheless, the global pigment market is expected to witness a healthy rise in the coming years (https://www.databridgemarketresearch.com/). The market value of natural pigments used as food colorants is predicted to reach USD 3.5 billion at 12.4 CAGR by 2027 (https://www.alliedmarketresearch.com/food-color-market). The market value of carotenoids alone is expected to reach USD 2.0 billion by 2026 (https://www.marketsandmarkets.com/Market-Reports/carotenoid-market-158421566.html).

Although pigment-producing microbes are ubiquitous, stressed (physical, chemical, and biological) environmental niches have more prevalence. For instance, microbial communities in cryo-environments produce myriads of pigments (Rehakova et al., 2019; Dhakar & Pandey, 2020; Sajjad et al., 2020). Microbial pigments such as carotenoid, melanin, violacein, and flexirubin have been isolated and identified from diverse cold niches (Vaz et al., 2011; Liu et al., 2019; Kumar et al., 2021). The bacterial carotenoids are one of the most reported pigments from variable cold habitats like a glacial fjord, Caspian sea, Antarctica, Italian alpine glaciers, and Himalayan niches (Reddy et al., 2003; Amaretti et al., 2014; Afra et al., 2017; Singh et al., 2017; Pandey et al., 2018). High-altitude Himalayas hosts various extreme niches harboring a range of stress conditions, including permafrost, freeze-thaw, oxidative stress, limited nutrients, and high UV (Stres et al., 2013; Kumar et al., 2021). Different microbial communities inhabit the hostile environmental conditions of such niches (Kumar et al., 2018a, 2022; Thakur et al., 2018), providing tremendous opportunities for bioprospecting pigment-producing microbes.

This review is focused on microbial pigment production, its ecological importance, and also presents a Himalayan perspective. Further, we discuss the importance of microbial pigments for studying microbial responses to changing environments, rapidly rising industrial interests, and potential applications. Finally, the biotechnological strategies for large-scale production are also discussed.

## Physiological and Ecological Significance of Pigments in Cold Adaptive Microbes

Microbes from the cold regions produce a variety of pigments as secondary metabolites in response to changing physiological and environmental signals and survival strategies (Quesada et al., 1999; Mueller et al., 2005; Dieser et al., 2010; Sajjad et al., 2020; Silva et al., 2021). The pigments are synthesized in harsh conditions to protect the microbial cells from excessive UV, photo-damage, fluctuating salinity, freeze-thaw cycles, and low temperatures (Mueller et al., 2005; Kumar et al., 2021). In addition, pigments also provide competitive advantages to the microbial community while thriving under various types of biotic and abiotic stress environments (Morgenstern et al., 2015; Lozano et al., 2020).

The microbial pigments from the Himalayan bacteria demonstrated a wide range of biological applications, including UV tolerance, cytotoxicity, antibacterial, and antioxidant potential (Correa-Llanten et al., 2012; Lapenda et al., 2015; Kumar et al., 2021). Some recent studies have shown the UV-protective characteristics of microbial pigments, such as carotenoid, violacein, and melanin (Reis-Mansur et al., 2019; Solano, 2020; Kumar et al., 2021). Additionally, carotenoid production from Antarctic bacteria played an important role in modulating membrane fluidity to cope with low-temperature conditions. It also protects cell damage against freeze-thaw (Jagannadham et al., 2000; Dieser et al., 2010). Carotenoids also help the fungus to tolerate harsh conditions of strong sunlight and UV radiation (Sajjad et al., 2020). Likewise, melanin and secondary metabolites accumulate in cells under environmental stress conditions (Bhosale, 2004). However, the psychrotolerant strain *Sphingobacterium antarcticus* produces a high amount of carotenoid pigment when compared to the mesophile *Sphingobacterium multivorum* (Jagannadham et al., 2000). Similarly, natural food colorants such as phycobiliproteins from mesophiles were found to be heat sensitive, resulting in reduced stability at high temperatures (Dufossé, 2018). Thus, pigment production from psychrophilic microbes confers substantial ecological and physiological benefits at cellular and community levels.

## Pigment Producing Microorganisms: The Himalayan Perspective

### A Colorful World of Microbial Diversity in the Himalayan Niches

The high-altitude Himalayan region looks barren and devoid of life to the naked eye. However, it hosts an unprecedented, colorful world of microbes underneath it. The Himalayan region throws harsh, challenging, and diverse microclimatic conditions, ranging from arid lands to permafrost glaciers and glacial lakes. These niches in the trans-Himalayan region host multiple environmental stresses, that is, fluctuating temperature, extreme cold, frequent freeze-thaw, oxidative stress, high UV intensity, low oxygen, and scarce nutrient availability. On the contrary, pigment production is one of the strategies that provides survival and adaptational advantages to many microbes in stressed environments (Mueller et al., 2005; Dieser et al., 2010; Silva et al., 2021). Therefore, high-altitude Himalayan niches are a hotspot for exploring pigment-producing microbes. The above hypothesis is supported by the bioprospection and diversity studies on pigment-producing microbes from high-altitude Himalayan niches summarized in Table 1.

**Table 1.** Pigment-producing microbes from cold niches of high-altitude Himalayas

Pigment(s)	Microbes/phylum	Isolation source	Biological applications	References
Violacein, deoxyviolacein (violet color)	*Iodobacter* sp. PCH194	Bhoot ground kettle lake, Sach Pass, Himalaya, India	Antimicrobial, anticancer, and UV protecting properties	Kumar et al., (2021)
Red pigment	*Rhodonellum psychrophilium*	Pangong Tso Lake located in Leh Ladakh, India	Antibacterial, antioxidant, growth stimulating properties	Bisht et al., (2020)
Yellow color	*Flavobacterium* spp.	Laigu, Zepu, Renlongba, and Gawalong glaciers, Tibetan Plateau	Adaptation under low-temperature conditions	Liu et al., (2019)
Various pigments (pink, yellow, and orange)	-	Tirich Mir glacier, Hindu kush Himalaya	-	Rafiq et al., (2019)
Yellow, orange, brown, violet, and pinkish-red	Proteobacteria, Firmicutes, Actinobacteria, Bacteroidetes	Himalayan glaciers, Uttarakhand, India	Adaptation against cold temperature	Panwar et al., (2019)
Carotenoid (orange)	*Penicillium* sp.	Indian Himalayan region	Antimicrobial potential	Pandey et al., (2018)
Carotenoids (various colors)	Proteobacteria, Actinobacteria, Bacteroidetes, and Firmicutes	Yuzhufeng glacier, Tibetan Plateau	-	Shen et al., (2018)
Prodgiosin (red pigment)	*Serratia nematodiphila* RL2	Lahul and Spiti, Himalaya, India	Antibacterial activity	Gondil et al., (2017)
Carotenoids (yellow-orange)	*Sanguibacter suarezii* KK6, *Kocuria turfanensis* KK7, *Kocuria rosea* KK12, *Planococcus maritimus* KK21	Leh and Ladakh, India	Survival strategies in cold conditions	Kushwaha et al., (2014)
Various pigments	Firmicutes, alpha- and gamma-Proteobacteria, Actinobacteria	East Rongbuk glacier, Mount Everest	Adaptation against stress	Shen et al., (2012)
Yellow pigment	*Leifsonia pindariensis*	Pindari glacier, Indian Himalayas	-	Reddy et al., (2008)
Yellow, pink, orange	*Bacillus odyssey, Flavobacterium* sp*. Cryobacterium psychrophilum, Kocuria carniphila, Frigoribacterium* sp.	Puruogangri glacier, Tibetan Plateau	-	Zhang et al., (2008)
Violacein (violet)	*Janthinobacterium lividum* XT1	Xinjiang glacier, China	Survival strategies in cold conditions	Lu et al., (2009)

A relatively high percentage of pigmented bacteria was found in the high-altitude glacial niches (Zhang et al., 2008; Shen et al., 2012; Shen et al., 2018; Panwar et al., 2019). For example, pigment-producing bacteria were isolated from different depths of ice core from the Puruogangri glacier in the Tibetan Plateau (Zhang et al., 2008). A total of 1385 bacterial isolates were obtained from east Rongbuk glacier, Mount Everest in the Himalayas and out of which 84.9% were found pigmented (Shen et al., 2012). Further, the studies showed that culturable and pigment-producing bacteria's abundance were higher in the middle and sequentially lower in the upper and below the ice core. The high percentage of pigmented bacteria in the high-altitude glacial samples validated the adaptive role of pigments for the bacteria (Shen et al., 2012). Another study unveiled the culturable bacteria belonging to four phyla from the ice core samples of the Yuzhufeng glacier situated at 3800 masl in the Tibetan Plateau (Shen et al., 2018). The study revealed 89% pigmented bacteria from entire colonies, and the proportion increased from 79 to 95% with the depth of the ice core. Different colored bacterial colonies such as yellow (47%), reddish-orange (24%), orange (16%), white (11%), pink (2%), and brown (<1%) were obtained from the ice core (Shen et al., 2018). HPLC analysis showed that 40% of the pigments were α-carotene, followed by 28% diatoxanthin. Other pigments identified were β-carotene, fucoxanthin, peridinin, and zea/lutein. Pigmented bacteria were also isolated from soil, water, and ice samples from the western Himalayas in Uttrakhand, India, with varying altitudes from 2300 to 4500 masl (Panwar et al., 2019). Amongst, some of the bacterial pigments showed intense antioxidant activity. These extensive diversity studies showed the abundance of pigment-producing bacteria in the high-altitude glaciers.

### Characterization of Pigments from the Bacteria/Fungi

Apart from the extensive diversity studies, only a few were reported to isolate and characterize pigments from bacteria. For instance, a red pigment-producing *Serratia nematodiphila* RL2 was isolated from the cold desert of Lahaul valley (Gondil et al., 2017). The pigment identified as prodigiosin showed an antibacterial effect against various pathogenic bacteria. Another red pigment-producing bacterium *Rhodonellum psychrophilium* GL8 was isolated from a high-altitude lake, Pangong Tso, Leh, India (Bisht et al., 2020). The pigments were a mixture of prodigiosin and other related compounds and showed antimicrobial, antioxidant, and bioenhancer properties. Blue-violet color-producing bacteria were also discovered in the high-altitude Himalayas. For example, the violacein-producing psychrotrophic bacterium *Janthinobacterium lividum* XT1 was isolated from a glacier in Xinjiang, China (Lu et al., 2009). A unique eurypsychrophilic bacterium, *Iodobacter* sp. PCH194, capable of violacein pigment production, was isolated from the sediments of Bhootground kettle lake situated at 4200 masl in Sach Pass, western Himalaya, India (Kumar et al., 2021). The violacein pigment was a mixture of violacein and deoxyviolacein and had promising antimicrobial and anticancerous properties. The yellow-colored *Flavobacterium* spp. were isolated from Tibetian glaciers, and their genome possesses genes encoding for carotenoid biosyntheses, such as phytoene synthase, lycopene-β-cyclase, and β-carotene hydroxylase (Liu et al., 2019). Similarly, the abundance of genes/proteins involved in carotenoid biosynthesis was found in the whole-genome metagenomes of high-altitude Himalayan lake sediments (Kumar et al., 2022).

Carotenoids and their derivatives produced by high-altitude Himalayan fungi *Penicillium* sp. GBPI_P155 possesses antibacterial potential. It may be a defense strategy against other microorganisms (Pandey et al., 2018). Similarly, many fungi produce pigments as an adaptive measure to cope with stress conditions of low temperature, UV radiations, and oxidative stress (Pandey et al., 2018; Sajjad et al., 2020). It suggested that microbial pigments such as carotenoids play an essential role in adaptation to the stress environment of high-altitude Himalayas.

### Co-Production of Biomolecules with Pigments as Sustainable Bioprocess

Besides the fundamental research, the pigment-producing microbes from the Himalayas are goldmines for industrially relevant bioproducts vis-à-vis microbial pigments. Since the Himalayan regions are less explored, they could be a rich source for new and unique pigment-producing microorganisms. Thus, efforts are required to explore its hidden treasures. Our lab focuses on bioprospecting high-altitude Himalayan microbiomes for basic and applied research (Kumar et al., 2018a, 2019, 2020, 2021, 2022; Thakur et al., 2018; Ambika et al., 2022). The isolation and identification of various pigment-producing bacteria from the high-altitude Himalayan region were accomplished during the course.

**Fig. 2.**
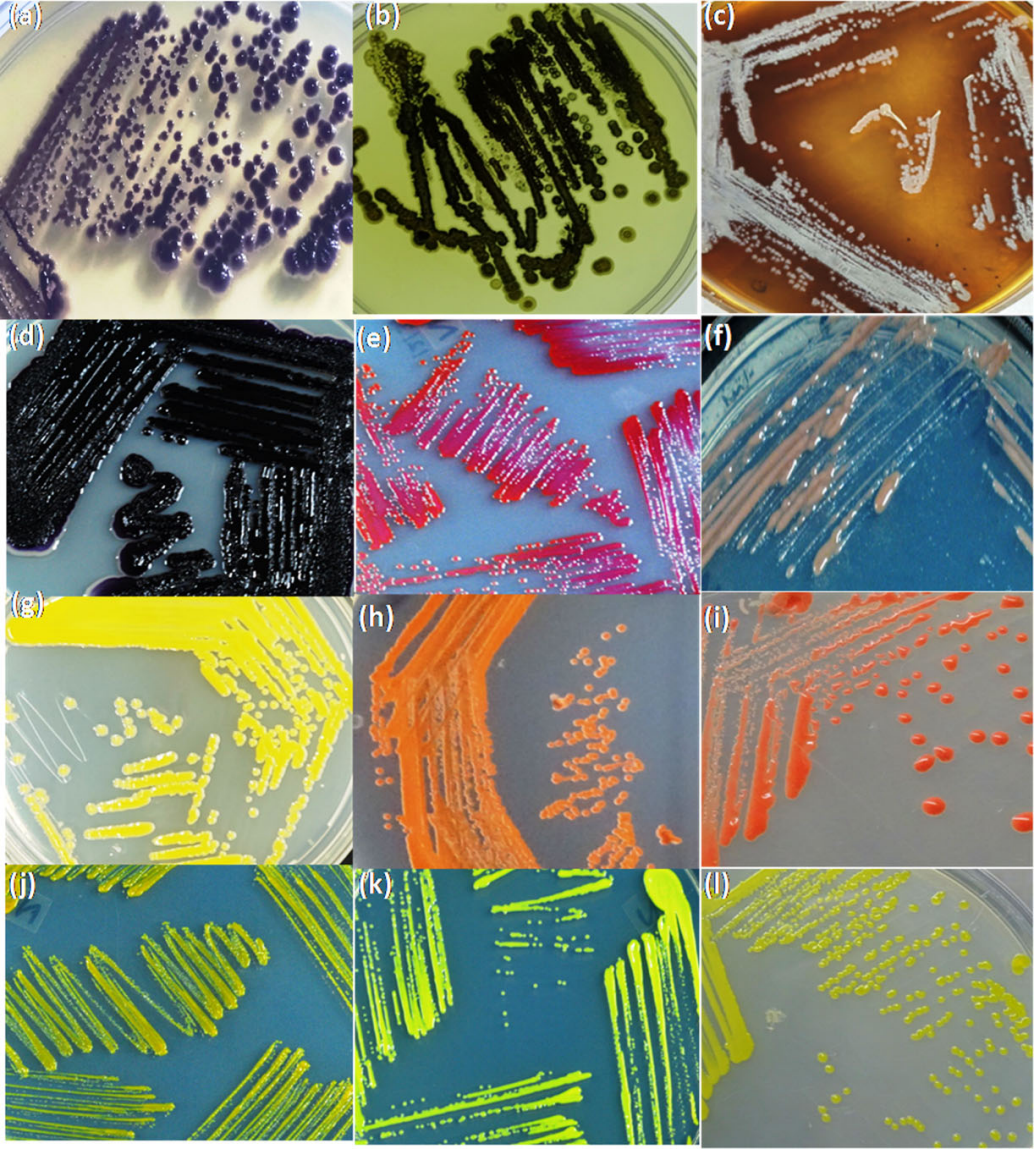
Pigment producing bacteria isolated from high-altitude trans-Himalayan region: (a) *Iodobacter* sp. PCH194, (b) *Streptomyces* sp. PCH436, (c) *Streptomyces* sp. PCH436, (d) *Janthinobacterium* sp. PCH410 (e) *Kocuria* sp. PCH206, (f) *Pedobacter* sp. PCH18 (g) *Pseudomonas* sp. PCH 413, (h) *Arthrobacter* sp., (i) *Bacillus* sp. PCH164, (j) *Flavobacterium* sp. PCH19, (k) *Arthrobacter* sp. PCH30, and (l) *Leifsonia* sp. PCH178.

A few prominent pigment-producing bacteria viz., *Iodobacter* sp. PCH194 (CP025781), *Kocuria* sp. PCH206 (MH096001), *Bacillus* sp. PCH164 (MF774150), *Pedobacter* sp. PCH18 (KY628836), *Flavobacterium* sp. PCH19 (KY628837)*, Arthrobacter* sp. PCH30 (KY628848)*, Leifsonia* sp. PCH178 (MF774164) (Kumar et al., 2018a, 2021; Thakur et al., 2018), *Pseudomonas* sp. PCH413 (MF774129), *Streptomyces* sp. PCH436 (ON080900), *Streptomyces* sp. PCH437 (ON080901), and *Janthinobacterium* sp. PCH410 (MZ396632). (Unpublished data) are shown in Fig. 2. Amongst, *Iodobacter* sp. PCH194 was successfully demonstrated for the pilot-scale production of violacein pigment and polyhydroxybutyrate as a co-product (Kumar et al., 2021). The patent for the Himalayan *Iodobacter* sp. PCH194 bioprocess for co-production of polyhydroxybutyrate and violacein pigment has been filed (Kumar et al., 2021). A few others are also being investigated in our lab.

## Biotechnological Strategies for Microbial Pigments Production

Bioprocess development is the key to the large-scale production of microbial pigments. It includes the up-scale production of microbial pigment followed by downstream processing. Several studies have developed bioprocesses for pigment production, such as carotenoids, flexirubin, violacein, and prodigiosin at ≥1.0 L level (Table 2). Most studies employed wild microorganisms in batch or fed-batch processes for pigment production. For example, carotenoid production was reported from *Rhodotorula glutinis* TISTR 5159, *Sporobolomyces roseus*, and *Sporidiobolus pararoseus* using cheaper carbon sources (Saenge et al., 2011, Petrik et al., 2014, Borba et al., 2018). Zeaxanthin, a type of carotenoid, was produced by the *Flavobacterium* sp. P8 strain in a 5 L batch bioreactor using yeast extract and peptone rich medium (Vila et al., 2020). However, the main problem associated with carotenoid production was the low yield. Flexirubin pigment was produced from *Chryseobacterium* spp. in a batch bioreactor with a yield of 0.2 and 0.52 g/L (Venil et al., 2015; Aruldass et al., 2016). Prodigiosin pigment production was attempted by employing *Serratia* spp. in a batch bioreactor. For instance, 18.2 and 8.0 g/L of prodigiosin were produced by *Serratia marcescens* strain CF-53 and UTMI in a 5 L stirred tank bioreactor using low-cost substrates like peanut oil cake (Naik et al., 2012) and brown sugar (Aruldass et al., 2014), respectively. Violacein pigment was produced using various wild types (Kanelli et al., 2018) and recombinant bacteria (Yang et al., 2011, Fang et al., 2015). Among the wild types, *Chromobacterium violaceum* was employed for large-scale violacein production using low-cost substrates (Aruldass et al., 2015). Engineered bacteria with violacein-producing genetic machinery further improve volumetric productivity over time (Yang et al., 2011; Fang et al., 2015; Niu et al., 2019).

**Table 2.** Biotechnological strategies for the up-scale production of bacterial pigments

Pigments	Substrates	Microbes	Strategy/Process	Scale (L)	Yield (g/L)	References
Carotenoids	Crude glycerol	*Rhodotorula glutinis* TISTR 5159	Batch bioreactor	1.0	0.135	Saenge et al., (2011)
	Corn steep liquor and sugar cane molasses	*Sporidiobolus pararoseus*	Batch bioreactor	1.5	0.0019	Borba et al., (2018)
	Spent coffee ground	*Sporobolomyces roseus*	Fed-batch bioreactor	12.5	0.0126	Petrik et al., (2014)
Zeaxanthin	Yeast extract, peptone, sodium chloride	*Flavobacterium* sp. P8 strain	Batch bioreactor	5.0	0.0312	Vila et al., (2020)
Flexirubin	Lactose, tryptophan and KH_2_PO_4_	*Chryseobacterium artocarpi* CECT8497	Batch bioreactor	50.0	0.52	Venil et al., (2015)
	Agro-industrial waste	*Chryseobacterium artocarpi* CEC8497	Batch bioreactor	5.0 and 50	0.54 and 0.20	Aruldass et al., (2016)
Violacein	Glucose, peptone	*Iodobacter* sp. PCH194	Batch bioreactor	7.5 and 10.0	1.5 and 1.25	Kumar et al., (2021)
	Glucose	Recombinant *E. coli* BL21 (DE3)	Batch bioreactor	5.0	1.75	Fang et al., (2015)
	Liquid pineapple waste and tryptophan	*Chromobacterium violaceum* UTM5	Batch bioreactor	50.0	16. 2	Aruldass et al., (2015)
	Tryptic soy broth	*Janthinobacterium* strain UV13	Batch bioreactor	5.0	0.077	Alem et al., (2020)
	Glycerol, meat extract, peptone	*Janthinobacterium lividum* 1522	Batch bioreactor	2.0	1.80	Kanelli et al., (2018)
Prodigiosin	Brown sugar (10%)	*Serratia marcescens* UTM1	Batch bioreactor	5.0	8.0	Aruldass et al., (2014)
	Glucose, glycerol	*Serratia marcescens* 02	Batch bioreactor	5.0	5.83	Tao et al., (2005)
	Peanut oil cake	*Serratia marcescens* CF-53	Batch bioreactor	2.0	18.2	Naik et al., (2012)
	Sucrose, peptone	*Serratia marcescens*	Batch bioreactor	7.0	0.39	Mohammed & Luti, (2020)
Amaranth	Mannitol and soybean flour + GAUSE'S medium	*Streptomyces coelicolor* MSIS	Batch bioreactor	5.0	9.0	Mohanasrinivasan et al., (2013)

Metabolic pathways for the biosynthesis of most of the pigments are complex. Therefore, metabolic engineering is usually tricky. Hence, alternative strategies such as cheaper substrates and co-production of multiple bioproducts must be explored. For instance, a simultaneous co-production strategy was developed in our lab using a Himalayan bacterium, *Iodobacter* sp. PCH194, which produced 1.5 g/L of violacein pigment and 10.0 g/L of polyhydroxybutyrate (Kumar et al., 2021). Similarly, astaxanthin-rich pigment and polyhydroxyalkanotes are simultaneously produced by *Paracoccus* sp. LL1 (Kumar et al., 2018b). Thus, the design of an appropriate cultivation system with a suitable bioreactor for industrial fermentation is required to achieve high production of pigments.

## Conclusion and Future Perspective

Microbes require specific features to produce biologically active pigmented compounds on an industrial scale. These include fast growth rates, scalability, high productivity, and preferably non-pathogenic. Additionally, the strains should include the utilization of low-cost substrates, ease for scale-up and downstream processing, high productivity, and overall low production cost. The microbial pigment should be non-toxic, stable, and tolerant to pH, temperature, and light. Bioprospecting pigment-producing microbes can obtain strains with desired features from extreme niches, including the high-altitude Himalayas, and further apply genetic engineering or strain improvement approaches to known potential microbes. The cryospheric microbes can synthesize natural colors as a protective shield against life-threatening ecological stresses. Therefore, new possible sources for pigment-producing bacteria must be investigated. Exploring microbial pigments from newer and extreme niches could provide novel and well-known pigment molecules for diverse industrial applications.

## Supplementary Material

kuac017_Supplemental_File
