# Supplementary figures and images for: Microbial pigments: learning from the Himalayan perspective to industrial applications
Source: J Ind Microbiol Biotechnol. 2022 Aug 6;49(5):kuac017. doi: 10.1093/jimb/kuac017 (PMC9559291; doi:10.1093/jimb/kuac017)

**Supplementary Figure**


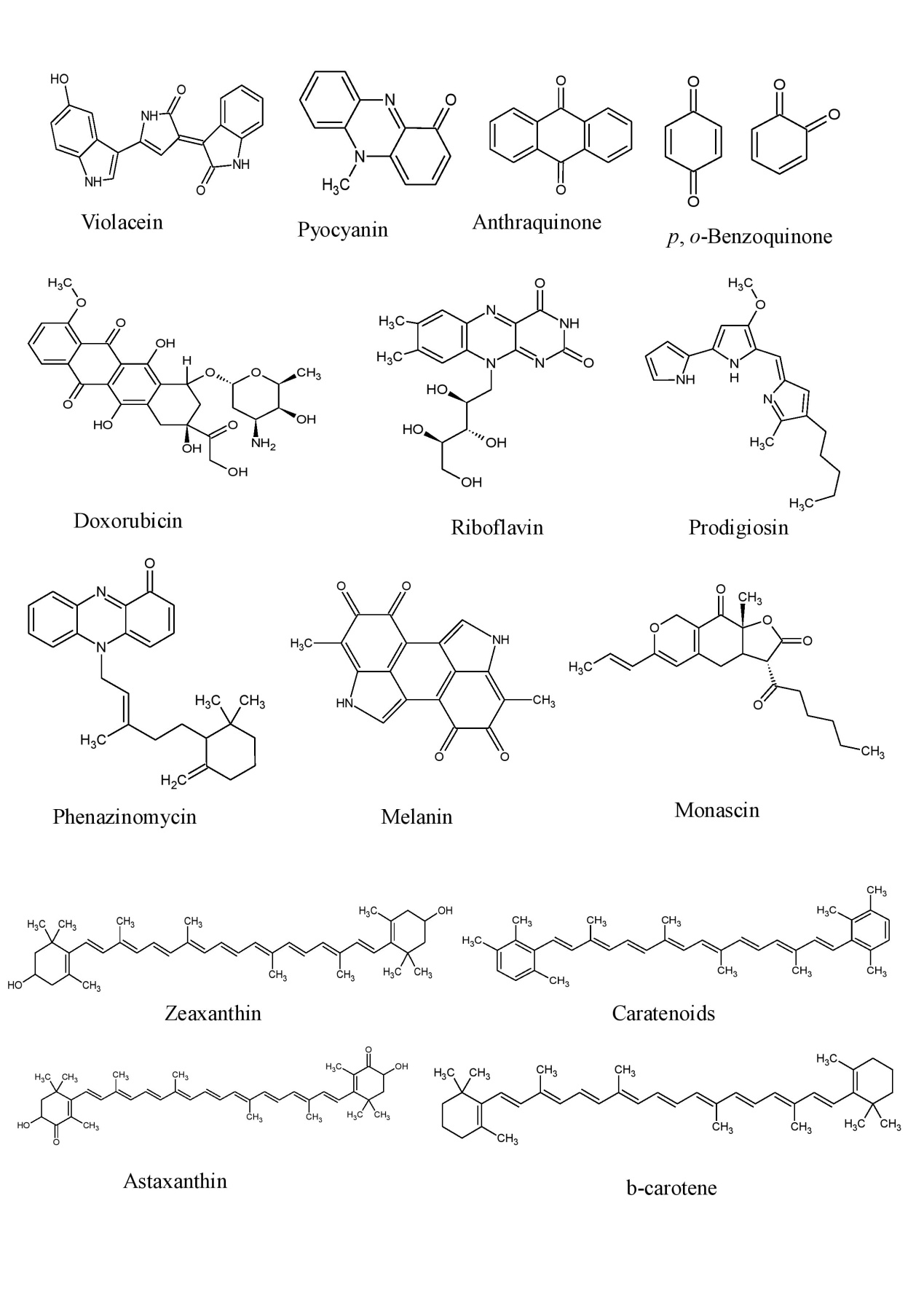


**Fig. S1** Structures for some of the important microbial pigments.

Supplement: kuac017_Supplemental_File [file kuac017_supplemental_file.doc]
